# Communicating Electronic Adherence Data to Physicians—Consensus-Based Development of a Compact Reporting Form

**DOI:** 10.3390/ijerph181910264

**Published:** 2021-09-29

**Authors:** Fine Dietrich, Andreas Zeller, Melanie Haag, Kurt E. Hersberger, Isabelle Arnet

**Affiliations:** 1 Pharmaceutical Care Research Group, Department of Pharmaceutical Sciences, University of Basel, Petersplatz 14, P.O. Box 2148, 4001 Basel, Switzerland; melanie.haag@unibas.ch (M.H.); kurt.hersberger@unibas.ch (K.E.H.); isabelle.arnet@unibas.ch (I.A.); 2 Centre for Primary Health Care, University of Basel, Rheinstrasse 26, 4410 Liestal, Switzerland; andreas.zeller@unibas.ch

**Keywords:** medication adherence, electronic health record, electronic monitoring, primary care

## Abstract

Information on medication adherence is missing in patient files, although it might be helpful to optimize treatment. An adherence report that presents data from electronic adherence monitoring and provides recommendations regarding pharmacological treatment could close this gap. We aimed to develop an adherence reporting form that combines suitable calculations and graphical representations to facilitate the physicians’ interpretation of (non-)adherence. Two consensus development panels were conducted. First, pharmacists with expertise in adherence monitoring debated the items needed to calculate and illustrate electronic adherence data. Second, physicians discussed the items they would need for an adherence report and were encouraged to propose new items. Preference was indicated by raising a green or red card. Voting was repeated until consensus was obtained. Third, first drafts of the adherence reporting form were created by two pharmacists. Seven pharmacists agreed on four metrics to express medication adherence and three graphical representations. Five physicians approved the four metrics and rated the dot chart as the most useful illustration for judging the patient’s adherence patterns. Additionally, they required a clinical–pharmaceutical evaluation of the adherence estimates considering drug-related properties. We developed an adherence reporting form for the first time in a compact format and based on the recommendations of experts. In addition, we considered the preferences of physicians, who appreciated the clarity of the reporting form.

## 1. Introduction

Adherence to medication is a key element for therapy success. According to Vrijens et al., medication adherence is described as “the process by which patients take their medication as prescribed” [1]. Non-adherence has been well described to cause hospitalizations, worsening of chronic disease, and increasing health care costs [2]. Several methods exist to identify patients at risk for non-adherent behavior. A standardized method exists with patients enrolled in the Swiss HIV Cohort, who must answer at every visit, the question: “How often did you miss a dose in the last 4 weeks?” [3]. Adherence can be measured with various methods, whereby electronic monitoring represents the current gold standard [4]. It is superior to other frequently used measures (e.g., pill count or dispensing data) as it delivers a nuanced picture of the individual intake pattern for each patient [5,6]. Thus, with the emergence of electronic monitoring data, the delivery of composite adherence evaluation is possible including elaborated comments. However, different ways to calculate adherence from electronically recorded intake data have been proposed in the literature [1,7] with mitigated success. Under these circumstances, clinicians are best placed to decide on the metrics most appropriate for their daily practice.

Unfortunately, patient files are often lacking information on medication adherence regardless of paper-based or electronic health records (EHR) [8,9]. For general practitioners (GP), an objective assessment of the intake behavior of their patients is almost impossible, rendering the outcome of any intervention very uncertain. Thus, the famous quote: if you cannot measure it, you cannot improve it [10] retains all its validity. As patient generated data, medication adherence could be integrated in EHR and be helpful to guide and adjust treatment [11]. With accurate knowledge of the medication intake behavior, a more patient-centered approach is possible to identify and optimize non-adherent behavior [5,6]. No standards are currently available on how to report the patient’s adherence data to health care professionals, with some isolated examples cited in the literature [12,13].

Wang et al. used a report based on smartphone application data (Corrie, ©Apple CareKit) targeting patients recovering from acute myocardial infarction [13]. A value of taking adherence for each medicine and a heat map showing missed doses or fractional taken doses are streamed on a daily basis into the physicians EHR. Besides its many strengths, one disadvantage of this application is that the results are not evaluated and no threshold is defined to link (non-)adherence to clinical outcome. Another promising project is the adherence program implemented at the University of Lausanne, Switzerland [12]. A report based on electronic monitoring data intends to support continuity of care in HIV therapy. The report consists of the summarized taking adherence, a calendar with the number of doses taken each day, a diagram displaying each intake time, and a heat map that highlights missed doses. The report is sent by the community pharmacist to the physician and nurse via email, where it must be integrated into the patient file, which is an inconvenient step at the GP’s backend.

This study aimed to develop a compact (one-page) reporting form for patient adherence data that were electronically recorded. It should combine suitable calculations and graphical representations so that non-adherence to polypharmacy can be easily interpreted by physicians. In addition, the integration into physicians’ EHR should be technically easily to perform.

## 2. Materials and Methods

We conducted two consensus development panels to incorporate the experts’ recommendations (pharmacist panel) and the preferences of the physicians (physician panel). A high amount of interaction between participants is beneficial for consensus finding. We considered all participants’ opinions and incorporated them into the results. The moderator had the role of presenting evidence and facts and structuring the discussion [14]. Voting was only used in the case of disagreement. Pharmacists and physicians were searched in the network of the Pharmaceutical Care Research Group, Department of Pharmaceutical Science, University of Basel. We defined experts as individuals who had experience and knowledge in adherence monitoring or/and practical experience in conducting studies that investigate medication adherence. There were no conflicts of interest on either panel or within the research team.

### 2.1. Pharmacist Panel

Pharmacist researchers in academia and practitioners from our network debated aspects of calculating and illustrating electronic adherence data. Each participant prepared topics within their practical field of experience and presented them to the group. Different adherence metrics and illustrations were discussed until a set of necessary metrics and possible illustrations was agreed upon. The final elements were placed on a flipchart to build a picture.

### 2.2. Physician Panel

Hospital physicians and GPs from our network debated the adherence calculations and representations of electronically measured adherence data that they would need for an adherence reporting form. A semi-structured scenario was prepared for a one hour discussion. The methods used by the physicians to assess the adherence of their patients in daily medical practice were collected. The physician participants were shown electronic adherence monitoring data of illustrative patients and were asked to estimate the medication adherence. Participants discussed the adherence calculations and graphical representations that adherence experts had agreed to beforehand. New proposals were included in the discussion. The physician participants indicated their preferred option by raising a green or red card. Voting was repeated until consensus was obtained (majority approval, three votes out of five). In a final step, the components of the report agreed on were arranged on a flipchart according to the participants’ preferences. The remuneration of the participants was CHF 100. The session was recorded and transcribed verbatim.

### 2.3. First Draft of the Reporting Form

The first draft of the adherence report was generated by two pharmacists (F.D., I.A.) and sent to the physician panel participants for feedback. We finalized the reporting form according to the obtained comments.

The Swiss software company openmedical AG programmed the final reporting form in their software solution mednet (App version 2.4.389, ©2016–2021 novcom AG) [15]. Mednet enables electronic data exchange between the GP and other health care providers (e.g., laboratories). Reports are automatically encrypted, signed, and sent through a secured channel to the openmedical platform (i.e., openmedical server). Received documents are automatically downloaded from the openmedical platform through a secured channel, decrypted (with signature check), and stored in the patients’ electronic file.

### 2.4. Data Analysis

Data analysis and generation of dot charts was performed with Microsoft^®^ Excel^®^ 2016. The adherence reporting form was generated with Microsoft^®^ Word^®^ 2016. The physician panel discussion was recorded using the App Linfei Recording© 2020 on a tablet computer. Transcriptions were inductively coded and grouped into themes by F.D. Results are presented as a summary of the panel transcript. Quotes are given in English after translation by F.D. from German, the original language. We report the means with standard deviations (SD) and absolute numbers of votes.

### 2.5. Ethic Statement

No patient-related data were collected in this study. Thus, the study did not fall into the Swiss human research act, and no ethics approval was required.

### 2.6. Patient and Public Involvement

No patient or public involvement because this study was focused on health care professionals.

## 3. Results

### 3.1. Pharmacist Panel

On 16 September 2019 for 4 h, seven pharmacists (57% female, mean age: 37 years ± 12) with various working environments participated in a panel discussion (Table 1). They agreed on four estimates to express adherence patterns. Taking adherence [expressed in %] represents the quotient of the number of doses effectively taken and the prescribed number of doses during the monitored period. Timing adherence [expressed in %] represents the quotient of the number of doses effectively taken within a pre-set time window, the so-called grace interval, and the prescribed number of doses during the monitored period. Correct dosing days [expressed in %] represents the quotient of the number of days with the correctly taken number of doses and all monitored days. A drug holiday [expressed in days] was defined as the number of consecutive days without medication intake and superior to 72 h.

**Figure 1:**
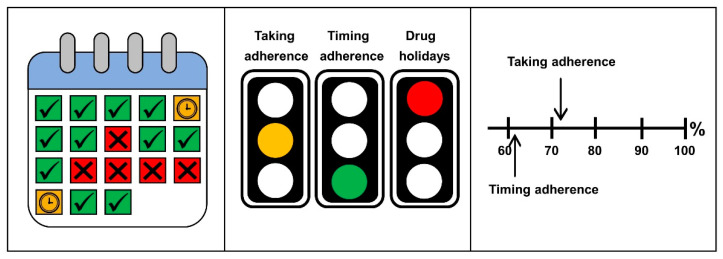
Graphical representations of medication adherence estimates in form of a calendar (**left**), traffic lights (**middle**), and numerical scale (**right**). The representations describe the intake of rivaroxaban once daily for the duration of 18 days, with the following estimates: taking adherence: 72%, timing adherence: 61%, drug holiday: 4 days.

Three graphical representations were selected by the panel (Figure 1). The calendar gives an overview of the correct dosing days of the monitored period. With this representation, each day was evaluated individually and no average values were indicated. A correct dosing day is labelled with a check mark; days with delayed intakes are given with a clock, and days with missed intakes with a cross. The traffic light system was chosen for its quick overview with intuitive interpretation. The numerical scale was the preferred option because threshold values were not needed for the visualization.

### 3.2. Physician Panel

Out of nine physicians, five of them (56% attendance, 20% female, mean age: 49 years ± 12) with various working environments (Table 1) participated on 17 January 2020in a panel discussion. One moderator (F.D.), two assistants (I.A., J.P.R.), and three observers (K.E.H., V.G., D.M.) were also present. The physicians already used different methods to assess the medication adherence of their patients in their daily medical practice. All stated that the interview and taking patients’ history were the most important sources of adherence assessment.

*“(…) we have the anamnesis. The patient statements, which I trust.”*

(P2)

Two participants used clinical outcomes such as blood pressure to evaluate medication adherence. Four other methods were reported: plasma level determination, electronic monitoring, refill data, and directly observed ingestion (for narcotic drugs).

**Figure 2:**
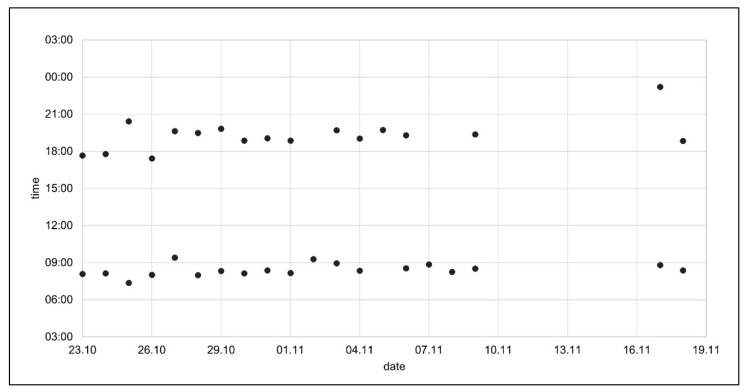
Dot chart displaying the electronically monitored medication adherence data of a twice daily regimen of apixaban over four weeks.

When asked to assess the adherence from a diagram displaying each intake time from an illustrative patient (Figure 2), all physicians made an estimation of the intake behavior. The most obvious deviation was the intake break of seven days (drug holidays), which was suspected to either be vacations, forgotten monitoring device, or a prescribed pause due to dental surgery. Three participants noticed that the evening intakes had a greater fluctuation and more missed doses compared to morning intakes.

#### 3.2.1. Adherence Calculations

The four metrics proposed to describe adherence were approved by a majority of physician participants (number of “yes” votes/number of all votes): taking adherence (4/5), timing adherence (3/5), correct dosing days (4/5), and drug holidays (5/5). Two participants proposed the use of timing adherence only for drugs that require a stricter monitoring or are unforgiving such as DOAC or specific antiepileptic drugs.

*“(…) after all, there are only a few drugs that you have to take at an exact time, and then it is important to me that he takes it.”*

(P3)

The correct dosing days was a source of discussion because it was considered too strict by two physician participants. Because an incorrectly dosing day applies independently of the number of doses per day, information is undifferentiated, that is, independently if a drug is to be taken several times a day or only once-daily. On the other hand, it was recognized that correct dosing days was stricter than taking adherence, and allows to reveal if a patient has difficulties with multiple daily intakes. All agreed that the metric drug holidays was mandatory.

**Figure 3:**
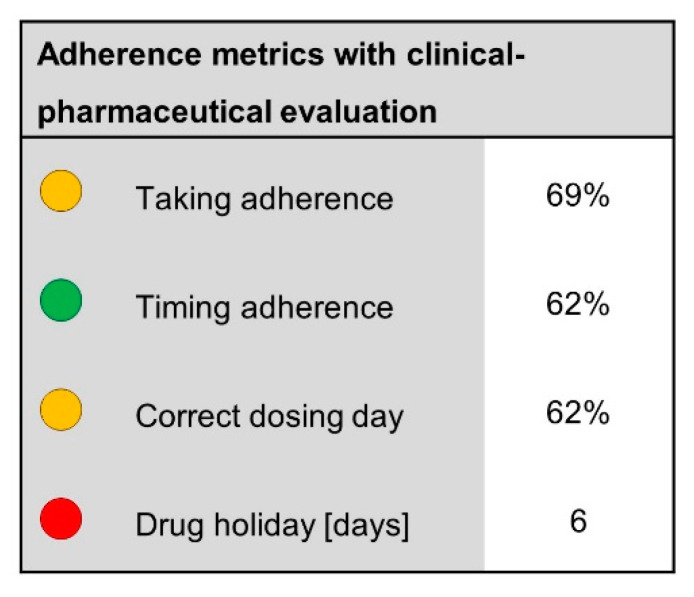
Adherence values with clinical–pharmaceutical evaluation; the values are depicted as circles with traffic light colors (green: satisfactory, yellow: unsatisfactory, red: critical).

Three participants expressed the wish to have a clinical–pharmaceutical assessment of the potential risk related to the drug in the case of unmet adherence. It was agreed that the calculated values in the reporting form should be displayed with traffic light colors (Figure 3). In the absence of specific cut-off values for each color, the evaluation should be evidence-based guided by pharmacological expertise and clinical judgement.

#### 3.2.2. Graphical Representations

The three proposed graphical representations (Figure 1) were rejected by the physician participants. However, the traffic light concept was considered promising to quickly identify a deviant behavior. The participants agreed that the dot chart was the most useful illustration for judging individual adherence patterns (Figure 2). One participant (P4) suggested displaying the grace interval into the dot chart, in order to facilitate the evaluation of timing adherence. Furthermore, it was decided to mark missed intakes in the dot chart.

#### 3.2.3. Reporting Form Structure

The document header includes patient age and sex, monitoring period, monitoring device number, date of the report, and initials of the pharmacist. One participant (P4) mentioned the need to indicate in the report the presence of vacations or hospital stays during this time as a source of variance in the usual medication taking behavior. The participants agreed that this information must be added in the header of the report.

**Figure 4:**
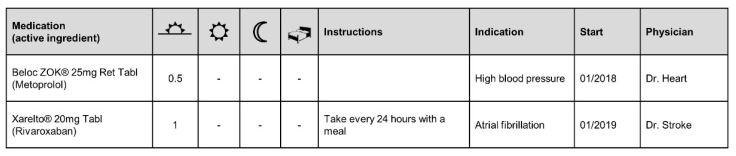
Illustrative medication chart of the adherence reporting form with icons for the time of the day.

A medication chart must be included in the report and contains the patient’s current and complete medication (Figure 4). It should be indicated whether one of the drugs on the chart is particularly critical in comparison to the others. This information was included in the title of the dot chart so that it is noticed immediately. The interpretation and recommendation were placed at the end of the reporting form. The physician participants were interested in obtaining a pharmaceutical assessment of potential risks related to non-adherence, even when the final interpretation remains in the physician’s responsibility.

*“I have to interpret this myself anyway, if this (forgotten tablets) is serious or not.”*

(P5)

### 3.3. First Draft of the Reporting Form

Two one-page adherence reporting forms were generated by two pharmacists (F.D., I.A.) and sent to the physician panel participants for feedback. Three (60%) responded with comments. All agreed that the reporting form was very well structured. They proposed minor graphical changes such as less dominant marks in the dot chart for missed doses.

*“What bothers me are the bars (…) they are a bit too massive for me (…).”*

(P3)

**Figure 5:**
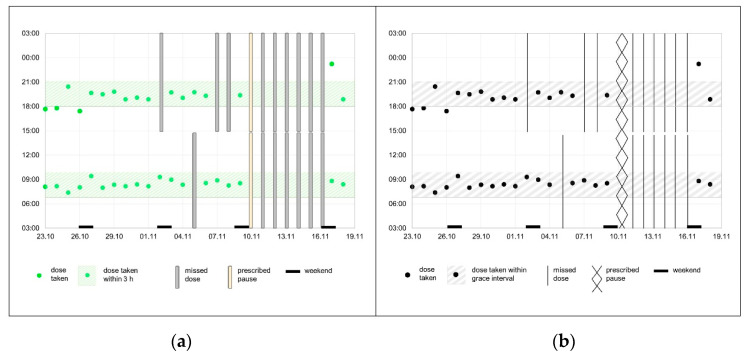
Two dot charts with electronic monitoring data (see Figure 2): (**a**) first draft after the physician panel, (**b**) after physician feedback.

In the final reporting form, the grey shaded bars from the first draft of the dot chart (Figure 5a) were replaced according to the physicians’ feedback by fine dark vertical lines, which were less disturbing (Figure 5b). See Supplementary Figure S1 for an example of the final adherence reporting form.

Our adherence reporting form was programmed to be electronically transmissible to the physician’s office using the mednet software of the company openmedical AG. Data protection is guaranteed, and the continuity of the process is simplified as no other step is needed to save the report in the patient file.

## 4. Discussion

To our knowledge, we have developed a compact (one-page) adherence reporting form that can be integrated into electronic health records. For the first time, the content and design were based on the preferences of the physicians as the final users. The one-page report is clearly arranged and contains calculated adherence values, a dot chart, and a pharmaceutical interpretation of the medication intake pattern.

We chose the method of the consensus development panel [14] because it is very effective in reaching consensus in a reasonable time. The interaction between the participants was crucial to achieve the discussed results. Although the participants in the panel discussion were practicing in the region of Basel, Switzerland, transferability to other settings is linked to specific conditions such as similar resources (staff) and IT infrastructure. However, the report can also be sent by email, fax, or postage service, which enlarges the scope of potential users.

We have deliberately avoided the distinction between adherent and non-adherent individuals. Many studies still use arbitrary cut-off values regardless of the prescribed medication. The definition ≥ 80% of doses taken is very commonly used to describe adherent patients [16]. However, a systematic review by Baumgartner et al. revealed that generalized adherence thresholds are questionable and only justified in connection with clinical relevance [17]. With the traffic light system, a continuous assessment of adherence was used to avoid dichotomous distinction. There were no fixed threshold values for the colors green, yellow, and red because the fixed values thresholds are not available from the summary of product characteristics (SPC) for the majority of medicines. The decision (green, yellow, red) is made by a trained pharmacist with clinical experience and is based on the pharmacological properties of the active ingredient, the dose regimen, and patient-related factors such as severity of treated disease and comorbidities [18]. The definition of the grace interval (i.e., the tolerated interval between two medication intakes) is also drug-specific and based on pharmacokinetic and pharmacodynamic characteristics [18]. We claim that a drug-specific consideration must be made for all adherence calculations. Thus, the pharmacist must decide which length of a drug holiday puts the patient at risk, knowing that most drugs are dosed so that blood concentration takes a longer interval before dropping to sub-therapeutic levels [19]. An interruption of medication intake for a few days can lead to a rebound effect for some drugs (such as beta blockers [18]) or can be tolerated by the body, as was shown for amlodipine [20]. However, the exact duration of tolerable interruptions are not indicated in SCPs.

Several studies have shown that increased adherence improves clinical outcomes. A study observed that patients with little variation in the time of taking lipid lowering drugs were more likely to reach their LDL-cholesterol target values [21]. In other studies, the increased adherence to medication used to treat HIV [22], diabetes [23], or hypertension [24] improved associated clinical values. In this sense, recommendations for improving adherence such as simplifying therapy or setting an alarm clock are included in the “recommendations/findings” section of our reporting form.

The clinical pharmaceutical evaluation of the adherence is not yet standardized and case assessments might differ from each other for that reason. Individual adherence assessment can limit reproducibility and would therefore need to be tested in a validation process. This is largely due to a lack of data concerning which adherence values are required for a specific medicine to reach an optimal therapeutic outcome. For some substances such as HIV medication, it is known that high adherence values (>95%) are necessary to sufficiently reduce the viral load [22]. However, for most other substances, evidence is low and studies on this topic are missing.

The report presents four adherence metrics (taking adherence, timing adherence, correct dosing days, drug holidays), which are the most self-explanatory measures of intake behavior. Furthermore, standardization to a maximum of 100% eases the awareness of the value. However, for specific medicines such as antibiotics, the longest period of time during which the medication was taken correctly could be useful for an acute medical therapy.

### 4.1. Strengths and Limitations

Our work has several strengths. First, we sought the opinion of experts in the field of adherence monitoring to delineate the different components of our reporting form. In addition, the preferences of the target group who were hospital physicians and GPs were implemented in the final reporting form. Second, the pharmacist panel provided the physician panel participants with concrete elements to decide upon. By doing this, we accelerated the decision process, which is illustrated by a rapid consensus finding and few diverging views. Third, we overcame some criticized features of existing adherence reports, and developed an exhaustive estimation of intake behavior around four adherence estimates. In addition, our report was designed to be transmitted electronically into the physicians’ EHR system, which ensures data security. Fourth, our adherence reporting form is suitable for all drug classes and all drug formulations. In addition, estimates of adherence to polypharmacy are delivered, which is of elevated usefulness for a patient with multi-morbidity and on multiple medicines. Fifth, our reporting form includes a pharmaceutical evaluation of adherence. This allows for an appropriate and patient-individual interpretation of the intake behavior by the physician.

We acknowledge some limitations. First, the number of participants in the physician panel was five, which is at the recommended lower limit for this research method. A larger number of participants or a second panel may have led to different results and preferences. However, the preceding pharmacist panel contributed to narrow the exercise. Second, we cannot exclude the possibility of a selection bias, as the physician participants already had experience in adherence research. On the other hand, this experience may have helped them to quickly involve themselves in the topic and formulate precise ideas and individual preferences for the report. Third, only three out of five physicians provided their feedback on the final reporting form, which reduced the representability. However, we assume that the two silent physicians agreed with the developed report. Thus, complete feedback can be extrapolated.

### 4.2. Outlook

Currently, a feasibility study is being undertaken to evaluate the electronic transmission of the adherence report from pharmacy software into the physicians’ EHR with chronic heart failure patients (ClinicalTrials.gov Identifier: NCT04326101). Furthermore, we are currently surveying the acceptance of the report among physicians who were not involved in its development. If proven feasible and acceptable, this way of communicating adherence reports could be pioneering through its content and its form.

## 5. Conclusions

To our knowledge, we present an adherence reporting form for the first time in a compact format that is based upon the preferences of the physicians. Further studies on the reproducibility of the clinical pharmaceutical evaluation and the efficacy on therapy-related outcomes must follow.

## Figures and Tables

**Table 1:** Characteristics of the pharmacist and physician panel participants.

Pharmacists (N = 7)	Working Environment
A1	Academic Research, Teaching
A2	Community Pharmacy, Academic Research
A3	Medical Laboratory Academic Research,
A4	Pharmacy Associations, Academic Research
A5	Community Pharmacy, Academic Research
A6	Hospital Pharmacy, Academic Research
A7	Community Pharmacy, Academic Research
**Physicians (N = 5)**	**Working Environment**
P1	General Practice, Academic Research
P2	Hospital
P3	General Practice, Academic Research
P4	Hospital, Academic Research
P5	Hospital, Academic Research, Addiction Clinic

## Data Availability

The data presented in this study are available on request from the corresponding author. The data are not publicly available due to privacy reasons.

## Supplementary Materials

The following are available online at https://www.mdpi.com/article/10.3390/ijerph181910264/s1, Figure S1: Example adherence report.
