# Supplementary material for: Communicating Electronic Adherence Data to Physicians—Consensus-Based Development of a Compact Reporting Form
Source: Int J Environ Res Public Health. 2021 Sep 29;18(19):10264. doi: 10.3390/ijerph181910264 (PMC8507897; doi:10.3390/ijerph181910264)
Supplement: Supplementary file 1 [file ijerph-18-10264-s001.zip › ijerph-1360242-supplementary.pdf]

Universität Basel

Fine Dietrich, study coordination
Petersplatz 14, PO Box 2148, 4001 Basel, Switzerland
Tel: +4161 207 15 29, mail: fine.dietrich@unibas.ch

Adherence Report

**Date**	17. Mar 2021
**Initials**	FDi, IAr

**Patient ID**	001	**E-mail**	-
**Year of birth**	1967	**Sex**	female
**Monitoring Device No**	UB5-0211	**Monitoring period**	29. OKT 2020 – 22. NOV 2020
**Special circumstances**	COVID-19 pandemic		

Index medicine (active ingredient)	Schedule	Instructions
Entresto® 50 mg (valsartan, sacubitril)	0.5 – 0 – 0.5 – 0	Interval between intakes maximum 12 hours

**Adherence metrics with clinical-pharmaceutical evaluation**	
(unsatisfactory) Taking adherence (dose taken)	72%
(satisfactory) Timing adherence (dose taken on time)	72%
(unsatisfactory) Correct dosing days	36%
(satisfactory) Drug holidays [days]	0
Grace interval (acceptable time variation)	± 7.5% (± 1.5 h)
	actual/ideal
Monitoring period [days]	25/25
Dose taken	54/75

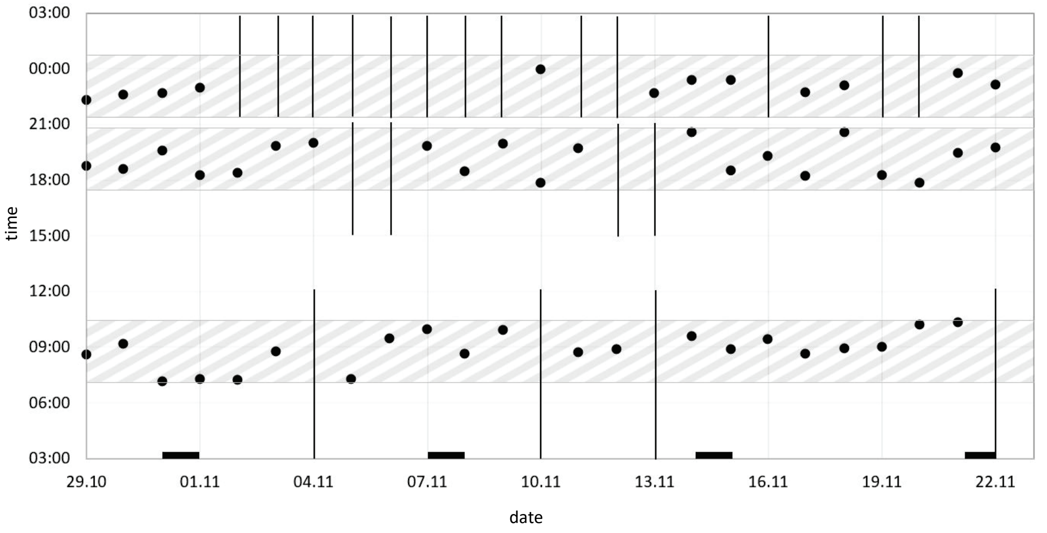

Adherence assessment	Recommendations
(satisfactory) **Intake times are regular:** small deviations from median intake time: morning 57 min, evening 52 min, night-time 30 min<br>(unsatisfactory) **21 missed doses:** 8 x Entresto® (+ co-medication), missed Zoldorm® doses are irrelevant<br>(satisfactory) **Intervals between Entresto® intakes** (morning – evening) are optimal: mean interval 9 h 59 min	Missed Entresto® doses might influence therapeutic goals.<br>A pillbox can be implemented to promote manageability of medication.

Legend

● dose taken | ● dose taken within grace interval | │ missed dose | ✕ prescribed pause | ▬ weekend | device defective | satisfactory (green) | unsatisfactory (yellow) | critical (red)

Co-medication (active ingredient)					Instructions	Indication	Start	Physician
Pantoprazole 40 mg	1	-	-	-	before meal	stomach protection	01/2018	Dr. A
Euthyrox® 0.125 mg (L-thyroxine)	1	-	-	-	before meal	thyroid	01/2018	Dr. A
Aspirin cardio® 100 mg (acetylsalicylic acid)	1	-	-	-	before meal	thrombosis prevention	02/2021	Dr. A
Gabapentin 100 mg	1	-	1	-		restless legs	01/2018	Hospital X
Bisoprolol 5 mg	0.5	-	-	-		heart failure	02/2021	Dr. A
Zoldorm® 10 mg (zolpidem)	-	-	-	0.5		sleep	01/2021	Dr. B
Paracetamol 500 mg					1 tablet every 6 hours if needed	pain	01/2018	-

© The formulas for the calculations are available upon request. Further use and reproduction only with written permission of the University of Basel.
